# Deconstructing the frame effect

**DOI:** 10.1167/jov.24.11.8

**Published:** 2024-10-10

**Authors:** Mohammad Shams, Peter J. Kohler, Patrick Cavanagh

**Affiliations:** 1 Department of Psychology, Glendon College, York University, Toronto, ON, Canada; 2 Centre for Vision Research, York University, Toronto, ON, Canada; 3 Department of Psychology, York University, Toronto, ON, Canada

**Keywords:** visual illusion, motion perception, object localization, motion-induced position shift

## Abstract

The perception of an object's location is profoundly influenced by the surrounding dynamics. This is dramatically demonstrated by the frame effect, where a moving frame induces substantial shifts in the perceived location of objects that flash within it. In this study, we examined the elements contributing to the large magnitude of this effect. Across three experiments, we manipulated the number of probes, the dynamics of the frame, and the spatiotemporal relationships between probes and the frame. We found that the presence of multiple probes amplified the position shift, whereas the accumulation of the frame effect over repeated motion cycles was minimal. Notably, an oscillating frame generated more pronounced effects compared to a unidirectional moving frame. Furthermore, the spatiotemporal distance between the frame and the probe played pivotal roles, with larger shifts observed near the leading edge of the frame. Interestingly, although larger frames produced stronger position shifts, the maximum shift occurred almost at the same distance relative to the frame's center across all tested sizes. Our findings suggest that the number of probes, frame size, relative probe-frame distance, and frame dynamics collectively contribute to the magnitude of the position shift.

## Introduction

The perceived position of an object is highly sensitive to the dynamics of its surroundings. For instance, a moving frame can generate the impression of motion on a static probe present inside it (Duncker, 1929; Johansson, 1950; Wallach, Bacon, & Schulman, 1978). The moving frame has an even larger effect when the probes are presented briefly (Cavanagh et al., 2022; Özkan, Anstis, Hart, ’t Wexler, & Cavanagh, 2021; Wong & Mack, 1981). In this so-called “frame effect,” the amount of position shift can be equal to the total distance the frame traveled, reaching values up to several degrees of visual angle. These offsets suggest that the probes’ locations are judged not in world coordinates but in the frame's coordinates (Özkan et al., 2021).

Position shifts can also occur in the absence of a moving frame. The perceived location of a stationary flashed object can be influenced by the presence of a nearby moving object (Eagleman & Sejnowski, 2007; Hubbard, 2014; Murai & Murakami, 2016; Whitney, 2002; Whitney & Cavanagh, 2000). For example, a stationary flashed object often appears to lag behind a coinciding moving object (Lappe & Krekelberg, 1998; Nijhawan, 1994). A somewhat larger shift is observed when an annulus rotates back and forth and probes are flashed at each motion reversal (Cavanagh & Anstis, 2013). However, these effects are notably weaker compared to the substantial position shifts induced by the moving frame, highlighting the unique strength of the frame effect in altering perceived positions.

Given the large magnitude of position shifts induced by the frame effect, an important question arises: What are the parameters that contribute to the size of the position shift in the frame effect? In this study, we examined the properties of the frame's effect on probes presented at various locations relative to the frame. Participants reported the perceived location after each trial by clicking on the location where they had seen the probe (Adamian & Cavanagh, 2024; Blom, Liang, & Hogendoorn, 2019). This method allowed us to investigate the magnitude and direction of the shift seen for each probe individually. In three experiments, we studied the influence of the number of probes (one vs. two) on the perceived shifts; we examined whether the effect accumulates over time by varying the number of back-and-forth motion cycles; we evaluated the relative contributions of motion before and after the probe flash; and finally we measured the position shifts as a function of the spatiotemporal distance between a single flash and a frame in continuous, unidirectional motion.

## Experiment 1: Number of cycles and probes in oscillating motion

Previous studies that investigated motion-induced position shifts typically asked subjects to report the location of a single flash (Adamian & Cavanagh, 2024; Cavanagh & Anstis, 2013; Eagleman & Sejnowski, 2000; Nijhawan, 1994) or to align two probes that flashed at the same time (Whitney & Cavanagh, 2000), but in the frame effect stimulus, the two probes flash in alternation. In a recent frame effect study (Cavanagh et al., 2022), a considerable percentage of participants reported that they saw the offset between the two probes only after a while. This suggests that it may take some time for the visual system to fully register the moving object and its trajectory to generate the frame effect.

To examine this, we varied the number of motion cycles of the frame and the number of flashed probes (one or two). In the One-probe condition, only one probe flashed either at the end of the rightward stroke or at the end of the leftward stroke whereas in the Two-probe condition, the two probes flashed in alternation at the end of each motion stroke. We defined location offsets as the horizontal offset between the location of the participant's mouse click and the physical location of the probe in the One-probe condition, and as the average horizontal offset between the two position reports and the probe location in the Two-probe condition (Figure 2A).

### Method

#### Participants

Twelve participants (eight females; one author) aged 18 to 36 years (*M* = 21.4; *SD* = 5.6) were from York University, Toronto, Canada. All participants other than one author were naïve to the purpose of the study and had normal or corrected-to-normal vision. The study was approved by the Human Participants Review Sub-Committee of York University's Ethics Review Board. Written, informed consent was obtained from each participant prior to their experimental sessions and participants were compensated with credit points. All methods of study were carried out in accordance with the Declaration of Helsinki guidelines and regulations (2003).

#### Apparatus

Stimuli were displayed on an LCD monitor with a resolution of 1920 × 1080 pixels, operating at a refresh rate of 60 Hz. However, the effective frequency was 30 Hz, as each frame was repeated twice. Subjects were instructed to restrain their head movements and to keep their viewing distance to the center of the display at 57 cm. The stimuli were generated and controlled with PsychoPy 2022.1.4 running in Python 3.8.

#### Stimulus and task

**Figure 1.**
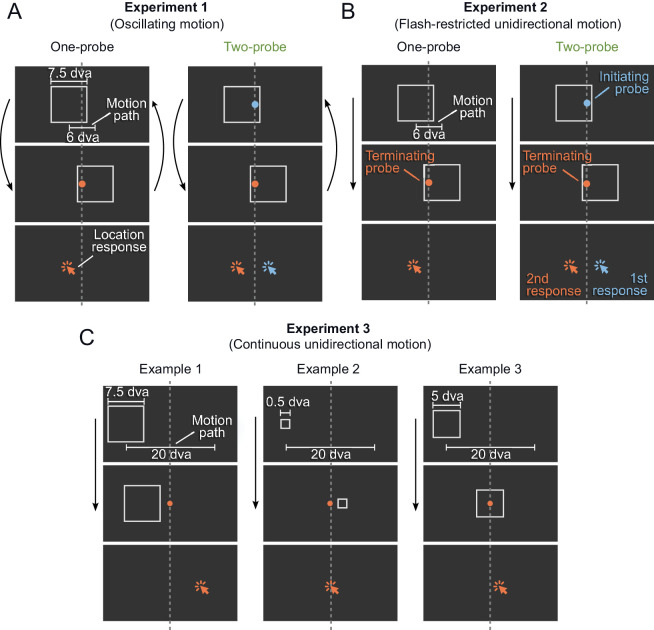
Stimulus design. (**A**) Experiment 1. A square frame moved back and forth for a variable number of cycles. The two end points of the frame's motion path are shown. In the One-probe condition, one probe flashed in a random color at one of the two end points of the motion path. In the Two-probe condition, a red probe flashed at one end of the motion path, while a blue probe flashed at the opposite end, and the participants could report their location in arbitrary order. (**B**) Experiment 2. Like A, but here the frame moved only in one direction, once. In the One-probe condition, the frame motion either started or ended with a flashed probe. In the Two-probe condition, the frame motion started after the first probe had been presented (initiating probe) and stopped before the second probe was presented (Terminating probe), and participants had to report their location in order of appearance. (**C**) Experiment 3. The frame moved over a long motion path once, and a single probe flashed at the middle of this motion path at a random time. The three examples depict three different frame sizes and three different relative position of the probe to the frame. The cursor was always the standard black and white shape. The colors of the cursor shown here are only for demonstration purposes.

Each trial started with the onset of the fixation mark, a plus sign (d = 0.7 dva), on a black background for a random duration (800–1200 ms). The fixation mark was presented 5 dva above the center for the observers would see the probes parafoveally because the effect can be reduced with direct fixation of the probes. To avoid any additional reference point beyond the frame itself, the fixation mark was then removed, but the participants were instructed to hold their fixation at that location throughout the trial. After a random duration of 300 to 700 ms, a frame appeared at a random position, −4 to −2 dva horizontally and −1 to 1 dva vertically off the center and moved back and forth horizontally (random first leg direction) within 433 ms over a path of 6 dva. The moving frame was an empty white square with a width of 7.5 dva and an edge thickness of 0.3 dva. The probes were filled circles (d = 0.5 dva) and appeared either in blue (RGB: 30, 144, 255) or red (RGB: 255, 99, 71), in an eight-bit intensity scale (Figure 1A). The number of cycles was either one, two, or three. In half of the trials, two probes flashed for 33 ms in alternation at the end of each path, during which the frame's motion was paused. The two probes appeared at the same screen location, but one was close to the right edge of the frame (when the frame was at the leftmost position of its motion path), and the other was close to the left edge of the frame (when the frame was at the rightmost position of its motion path). In the other half of trials, only one of the two probes flashed. Participants were instructed to maintain fixation on the location indicated by the fixation mark early in the trial and to report the location of the probe(s) with a mouse click (in arbitrary order if there were two probes) as soon as the mouse cursor appeared. Between 300 and 700 ms after the frame motion ended, the cursor (a typical arrow) appeared 8 dva below the fixation mark's previous location on the screen (3 dva below the frame's pathway), with no horizontal offset, allowing participants to freely move it in any direction (see Supplementary Video S1). There were six conditions (motion cycles, three values, 1, 2, 3; probe number, two values, 1, 2) for a total of 60 trials per participant (conditions presented randomly). The experiment lasted about eight minutes.

### Results

**Figure 2.**
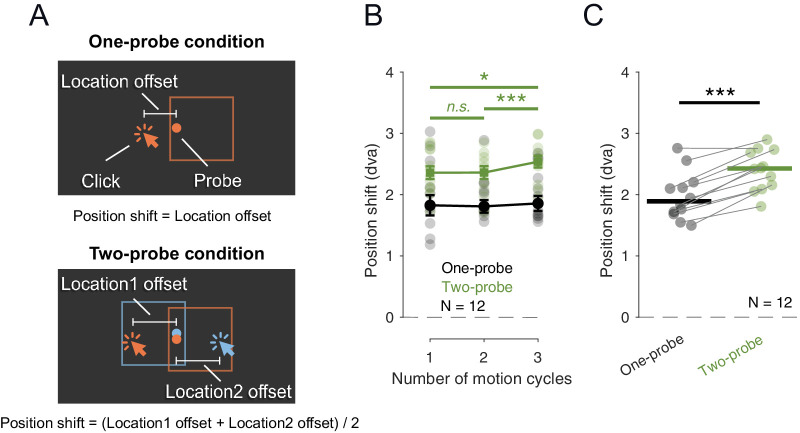
Experiment 1. (**A**) In the One-probe condition, the reported position shift was defined as the offset between the location response and the actual probe location. In the Two-probe condition, reported position shift was the average of the two position shifts. (**B**) Position shift as a function of the number of motion cycles, shown separately for One-probe condition (black) and Two-probe condition (green). Filled solid circles indicate the median across participants and error bars indicate ± *SEM*. Transparent circles indicate individual participant's data. (**C**) Same data as in B but collapsed across motion cycle numbers. Thick horizontal lines indicate median across participants and thin lines connect the two data points of each participant. ***p ≤ 0.001;  *p ≤ 0.05;  *n*.*s*.  *p* > 0.05.

As shown in Figure 2B, in the One-probe condition, the horizontal position offset did not vary as a function of the number of motion cycles (Friedman's test; χ^2^(2) = 0.167, *p* = 0.92). In the Two-probe condition, however, the position offsets did vary across motion cycles (Friedman's test: χ^2^(2) = 10.667, *p* = 0.005). The offset in the Three-cycle condition was 6.2% larger than in the Two-cycle condition (Wilcoxon signed-rank test: *W* = 1, *r* = 0.97,   *adjusted* *p* = 0.002, Benjamini-Hochberg correction for multiple comparisons) and 6% larger than in the One-cycle condition (Wilcoxon signed-rank test: *W* = 9, *r* = 0.77, adjusted *p* = 0.048).

In all three motion cycle conditions, the median location shift (from the physical location to the reported location) was larger in the Two-probe condition than in One-probe condition. To examine this further, we averaged the location responses of each participant across the three motion cycle conditions, separately in One-probe and Two-probe conditions (Figure 2C). The median difference in location responses between the Two-probe and One-probe conditions was 17% across participants, with responses in the Two-probe condition being 0.4 dva larger compared to the One-probe condition (Wilcoxon signed-rank test: *W* = 1, *p* = 0.001, *r* = 0.97). The position shifts between the One-probe and the Two-probe conditions were strongly correlated across participants (Kendall rank correlation: τ = 0.76).

Three motion cycles induced slightly larger position shifts than one and two motion cycles in the Two-probe condition but there was no effect of the number of cycles in the One-probe condition. Overall, we found that the reported position shifts in the Two-probe condition were larger than in the One-probe condition.

## Experiment 2: Number of probes in unidirectional motion

Several studies have pointed out that motion after a flashed probe is more effective in shifting the probe's perceived position than motion before the probe (Blom et al., 2019; Cavanagh & Anstis, 2013; Eagleman & Sejnowski, 2000; Takao, Sarodo, Anstis, Watanabe, & Cavanagh, 2022). In previous frame effect studies, the frame typically moved back and forth until the participant reported the perceived offset. In such a procedure, each probe has motion before and after its presentation so that the contributions of motion before and after the probe cannot be evaluated separately. In this experiment, there was only a single transit of the frame with the flashes presented either at the start or the end of the motion, or both (Flash-restricted unidirectional motion). This procedure enabled us to investigate how much of the induced position shift is due to the motion before and how much to the motion after the probe. In the Two-probe condition, the first probe flashed when the motion began (Initiating-probe condition) and the second probe flashed when the motion stopped (Terminating-probe condition). In the One-probe condition, only one of these two probes flashed (Figure 1B).

### Method

After finishing Experiment 1, each of the 12 participants from Experiment 1 then started Experiment 2.

#### Stimulus and task

The task procedure here was similar to the one in Experiment 1 with two exceptions: First, the frame moved only for half a cycle, i.e., in one direction, either leftward or rightward (selected randomly); second, when there were two probes, participants were instructed to click on each location sequentially, following their respective order of appearance (see Supplementary Video S2). There were three conditions (two probes, single initiating probe, single terminating probe) for a total of 30 trials per participant (conditions presented randomly). The experiment lasted about four minutes.

### Results

**Figure 3.**
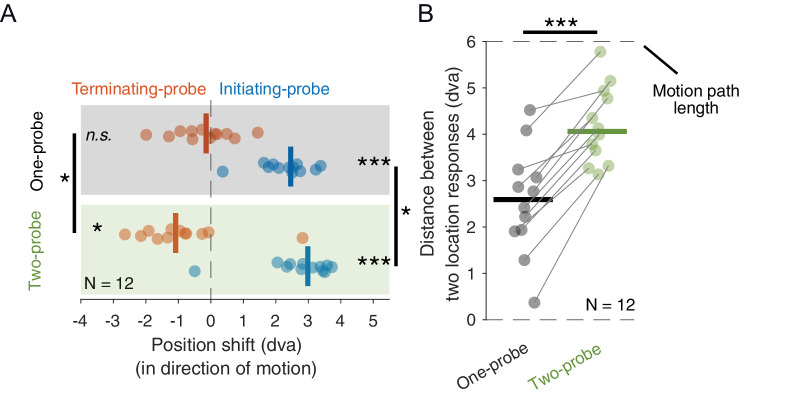
Experiment 2. (**A**) Position shifts of the terminating (orange) and initiating (blue) probes, shown separately for the One-probe (black box) and the Two-probe (green box) conditions. Each dot indicates the average position shift across trials of one participant in the specified condition. The vertical dashes indicate the median position shift across participants. (**B**) The distance between the initiating probe and the terminating probe for each participant in One-probe (black) and Two-probe (green) conditions. ***p ≤ 0.001;  *p ≤ 0.05;  *n*.*s*.  *p* > 0.05.

As demonstrated in Figure 3, in the One-probe condition (the black box), the reported position shifts of the terminating probes were not different from zero (Wilcoxon signed-rank test: *W* = 29, *p* = 0.47), but the reported position shifts of the initiating probes were 2.5 dva in the direction of the following motion (Wilcoxon signed-rank test: *W* = 0, *p* < 0.001, *r* = 1). In the Two-probe condition (the green box), the location offsets of the Terminating probes were shifted at median 1.1 dva in the direction opposite to the frame's preceding motion (Wilcoxon signed-rank test: *W* = 12, *p* = 0.034, *r* = 0.69), and the position shifts of the initiating probes were 3 dva in the direction of the following motion (Wilcoxon signed-rank test: *W* = 1, *p* = 0.001, *r* = 0.97).

As in Experiment 1, we observed that the position shifts were larger when two probes were presented. The position shifts of the initiating probes were 0.5 dva further in the direction of motion in the Two-probe condition than in the One-probe condition (Wilcoxon signed-rank test: *W* = 13, *p* = 0.042, *r* = 0.67) and the position shift of the trailing probes was 0.9 dva further in the direction opposite to the frame's motion (Wilcoxon signed rank test: *W* = 12, *p* = 0.034, *r* = 0.69).

To see the total distance between the initiating probe and the Terminating probe that each participant perceived, we calculated the distance between the two reported locations, separately in the One- and Two-probe conditions (Figure 3B). The median difference in the distance between the two location responses in the Two-probe condition was 71% larger than in the One-probe condition (reported in separate trials) across participants (Wilcoxon signed rank test: *W* = 0, *p* < 0.001, *r* = 1), with a median increase of 1.7 dva. As was the case in Experiment 1, the position shifts between the One-probe and the Two-probe conditions were here strongly correlated across participants (Kendall rank correlation: τ = 0.70).

As reported previously (Blom et al., 2019), the motion after the flash strongly shifted the perceived position of the probe in the direction of motion. However, motion before the flash significantly shifted the perceived position of the probe in the direction opposite to the motion of the frame, but this effect was observed only on trials where an initiating probe was also present. Additionally, as in Experiment 1, we observed that adding a second probe increased the total induced position shift of each probe, but this time the increase was much larger (71% vs. 17%).

## Experiment 3: Spatiotemporal profile and frame size in unidirectional motion

Probes that flash closer to a moving contour are shifted more than probes farther away (Cavanagh & Anstis, 2013; Whitney & Cavanagh, 2000). In Experiment 2, we focused on the temporal relation between the flash and the motion—whether the motion preceded or followed the flashed probe. Note that the temporal relation between flash and motion can also be interpreted as a spatial relation (Watanabe, 2005; Watanabe, Sato, & Shimojo, 2003)—with the probe ahead of (leading) the frame's center or behind (trailing). In this experiment, we asked how the position shift of a single probe varied as a function of the frame's size and the spatial relation between the probe and the frame.

**Figure 4.**
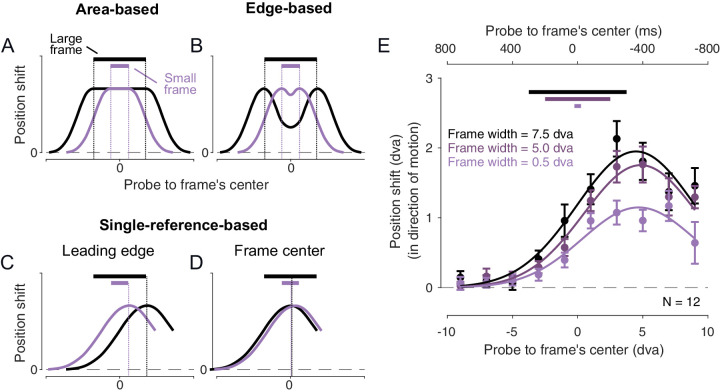
Experiment 3. Hypothetical and actual spatiotemporal position offset profiles. (**A**) Area-based model. Probes that flash at any location within the frame are shifted uniformly whereas the effect drops away outside the frame. (**B**) Edge-based model. Probes that flash near the edges of the frame are shifted the most. (**C**, **D**) Single-reference-based model. Probes that flash at a certain distance with respect to reference location in the frame undergo the strongest shift with the effect dropping away with distance from that location. This effective reference could be, for example, the leading edge of the frame (**C**) or the center of the frame (**D**). (**E**) The observed spatiotemporal profile of the position shifts. The lower abscissa indicates the spatial distance between the probe and the frame's center, and the upper abscissa indicates the equivalent temporal distance. The filled circles and the error bars show the average position shifts and the ± *SEM* across participants. The continuous curved lines are Gaussian fits with parameters reported in Table 1. The solid horizontal lines at the top indicate the widths of the three frames used in this experiment.

There are several ways the frame's components may affect the probe's perceived location. For example, the induced position shift could be uniform within the frame but drop away with distance from the frame (Figure 4A). Alternatively, both edges of the moving frame (Cavanagh & Anstis, 2013) may be responsible for inducing a shift with the effect dropping away from the edges on either side (Figure 4B). Finally, there may be one single location in the moving object that is the reference for the position shifts (Figures 4C, 4D). In this case, the maximum shift would occur for probes at this single reference with the effect dropping away with distance from this spot; for example, this single reference could be the frame's leading edge (Figure 4C) or its center (Figure 4D). As we consider these potential models, it is important to note that the locations of maximum effect are not necessarily aligned with the edges or the center of the moving object. For example, if the profiles in Figure 4A were shifted about 30% of the frame's width to the right, we would get a large shift for the probes near the leading edge and a small shift for the trailing edge. To distinguish these potential models, we measured the spatiotemporal profile of the effect of moving frames transiting along a long path (continuous unidirectional motion) with three different sizes by flashing a probe at different locations relative to the frame's motion path where again the participants reported the probe positions with mouse clicks.

### Method

After finishing Experiment 2, each of the 12 participants of Experiment 1 and Experiment 2 then started Experiment 3. The apparatus used in this experiment was identical to that in the previous two experiments.

#### Stimulus and task

Each trial started with the onset of the fixation mark, a plus sign (d = 0.7 dva), on a black background for a random duration (800–1200 ms), 5 dva above the center of the monitor. After a random duration of 300 to 700 ms, a frame of a random size (7.5, 5, or 0.5 dva, corresponding to an edge thickness of 0.3, 0.2, or 0.05 dva, respectively) appeared at a random position, −11, −10, or −9 dva horizontally and −1, 0, or 1 dva vertically off the center, moving over a path of 20 dva within 1433 ms. A single probe (d = 0.5 dva, RGB: 255, 99, 71 in an eight-bit intensity scale), vertically aligned with the midpoint of the frame, was flashed at the center of the frame's 20 dva path at a random time within the motion interval, ranging from 716 ms before and 716 ms after the frame's center reached the probe location. This was equivalent to a spatial horizontal offset that ranged from 9 dva ahead of the frame's center to 9 dva behind it (see Supplementary Video S3). A total of 180 trials were collected from each participant (frame size and probe location selected randomly on each trial). The experiment lasted about 20 minutes.

### Results

The response profile for each frame size was calculated by binning the horizontal value of each reported location (mouse click) in 2 dva bins from −9 to 9 dva relative to each frame's center. We then fitted a Gaussian function (*G*) to the resulting distribution:

$$G=\;Ae^{-\frac{1}{2}\;{\left(\frac{x-\mu }{\sigma }\right)}^{2}}$$

where *x* is the mean horizontal offset in each bin, *A* is the estimated maximum reported shift of the probe relative to its physical location, μ is the spatial distance between the probe and the frame's center at which the estimated maximum shift occurred, and σ is the standard deviation of the spatial profile of the reported probe locations. The data were fitted separately for each frame size.

The spatiotemporal profile of the position responses followed a Gaussian-like pattern (Figure 4E). The Gaussian fits (mean adj*R*^2^ = 0.93) to these data revealed that despite the large difference between the smallest and the largest frame size (7 dva in range), the peak offset occurred when probes flashed about 5 dva in front of the frame's center for all frame sizes. This offset is equivalent to about 400 ms of the frame's motion from the moment that the probe flashed until the frame's center moved over the flash location (Table 1). We also observed, that as the frame size increased, the amplitude of the position shift increased but its width did not.

**Table 1.** Experiment 3. Parameters of the gaussian fits.

		Peak shift distance (μ)		Standard deviation (σ)		
Frame width (dva)	Peak shift amplitude (*A*) (dva)	(dva)	(ms)	(in space)	(in time)	Fit quality (adj*R*^2^)
7.5	1.9	4.5	358	4.5	361	0.92
5.0	1.8	4.8	387	4.3	348	0.95
0.5	1.1	4.7	375	4.3	347	0.92

The peak position shift occurred for probes that flashed at certain spatiotemporal distance to the frame's center independent of the frame size. Although larger frames induced larger position shifts, the standard deviation of the distribution of the shifts was unaffected by the frame width. This result is a good match to the Single-reference-based model (Figure 4D) with the additional feature that the amplitude of the profile depends on the frame width.

## Discussion

Our goal in this study was to determine which parameters of the frame stimulus (Cavanagh et al., 2022; Özkan et al., 2021) contribute to the larger effect sizes in the frame effect compared to other motion-induced position shifts. In previous demonstrations, the frame effect had several characteristic properties including repetitive cycles of the frame motion, assessment of perceived offset between two probes, reversal of motion after each probe presentation, and presentation of the probes inside the frame.

We found that the apparent position shift induced by the frame effect exhibited minimal accumulation across repetitive frame cycles. The maximum change over cycles was an approximately 6% increase by the onset of the third cycle, and only when two probes were present. Consequently, it appears that the ability of the frame effect to elicit a large shift in position is only weakly affected by repeating cycles.

The number of probes, in contrast, had a strong impact on the apparent position shift. The separation reported between two probes when both were present on each trial was 71% greater than the separation between the probes when tested separately in the Flash-restricted unidirectional condition (Experiment 2, Figure 3B) and 17% greater when the probes were tested separately in the oscillating motion condition (Experiment 1, Figure 2C). Importantly, both the first and second probes exhibited increased shifts in opposite directions when an additional probe was introduced. This influence of each probe on the perceived location of the other probe suggests a localization mechanism that integrates spatial information over a time window that covers both probes, and retrospectively assigns relative locations to the probes.

Frames compete and observers may switch between multiple competing coordinate systems depending on the circumstances (Cavanagh et al., 2022). Therefore the reported locations are affected by the relative weight of the competing systems, specifically the moving frame itself, and the world coordinates, those of the static monitor and surrounding room. When there are two probes, their relative separation in frame coordinates dominates as they serve as reference points for each other. However, when there is only a single probe, its location in the surrounding static world coordinates takes more weight, reducing the perceived shifts. We speculate that weight of the world coordinates is even stronger for the Terminating-probe condition than in the Initiating-probe condition. In the Terminating-probe condition, the frame has stopped moving, and the location of the probe within the frame and its location in world coordinates match.

Previous work on the frame effect (Özkan et al., 2021) asked participants to reproduce the perceived offset between the two flashed probes while observing the stimulus. The participants reported an offset almost equal to the motion path length (100%). In our study, we asked the participants to report the location of individual probes with a mouse click after the disappearance the stimulus based on the remembered location of the probes. The maximum offset observed with this method was about 70% of the motion path length. In the offset-matching method, the match can be made as soon as both probes have appeared and the offset between the two probes is judged on its own. In the memory-based method, participants must establish the location of each probe in screen or world coordinates, keeping them in mind until the end of the trial. The conversion to screen coordinates may underlie the weaker effect observed in our study, but we emphasize that the memory-based approach nonetheless produces a strong frame effect.

In previous articles, the frame effect has been shown for probes at the reversals of an oscillating frame observed over multiple cycles (Cavanagh et al., 2022; Özkan et al., 2021) Here we demonstrated that a significant position shift occurs even when the probes are not at the reversals. In the first case, in Experiment 2, the probes were presented at the start or the end of a unidirectional motion (Flash-restricted unidirectional motion). The resulting separation between the two probes was smaller than that seen for the reversing motion case: The unidirectional motion effects were 32% less than for the reversing motion when the probes were tested separately and 12% less than when both probes were present, one at the start and one at the end of the single pass. However, this reduction in separation was due principally to the much smaller effect on the terminating probe, whereas the shift of the initial probe was as large as that for each of the individual probes in the reversing motion case. With reversing motion, the shift was large and in opposite directions for each probe such that the reversing frame appears to turn each flash into the equivalent of the initial flash of the unidirectional motion.

The second case with no motion reversals (Experiment 3), presented a single probe at various offsets relative to the frame and both inside and outside the frame. This configuration also produced a significant shift in apparent location, about 2 dva. This value cannot be compared to the path length in this case but in terms of absolute shift, it is comparable to those seen for the initial probe in the single pass motion (about 3 dva) and for the individual probes in the reversing motion (about 2.5 dva). Importantly, the largest induced shift occurred for probes about 4.5 dva ahead of the frame's center (Figure 4E). Larger frames produced larger shifts overall, but the location that produced the maximum shift was always the same with respect to frame's center. Interestingly, the maximum shift was found for probes flashed outside the frame and ahead of it. In previous studies, the frames were often larger (Özkan et al., 2021), and the probes was always inside the frame. This configuration placed the location of the probe at the optimal position, 4.5 dva, from the frame center.

The results of Experiment 3 closely matched the predictions of the single-reference-based model. It suggests that the visual system may rely on the center of the moving frame as a reference point, adjusting perceived positions based on the distance from this reference. This could be a mechanism that helps maintain spatial constancy and reduce ambiguity during passive viewing of dynamic visual environments or provide precise landmarks for retinal remapping during saccadic eye movement (Cavanagh, Hunt, Afraz, & Rolfs, 2010; Collins, Rolfs, Deubel, & Cavanagh, 2009; Deubel, Koch, & Bridgeman, 2010).

Overall, we found that the large effect size for the frame, as reported in previous studies, can be attributed to a combination of several factors: reversing motion, the presence of two probes per cycle, larger frame sizes, and probes positioned approximately 4.5 dva (350 ms) ahead of the frame's center. These factors contributed to the strongest position shifts observed, yet the underlying mechanism that drives the basic effect of the moving frame on the position of the static flash remains unclear. Future research may explore the distinct contributions of memory-based processes and spatial integration over time, particularly in scenarios involving multiple probes. Additionally, investigating the impact of different response methods, such as offset-matching versus memory-based clicking, could provide further insights into the observed effects. Finally, the potential dissociation of time and space in the frame effect warrants continued study, for instance, by manipulating the frame's speed to determine whether faster-moving frames result in larger position shifts (time dependent) or the same shift (distance dependent). Such investigations would further clarify the unique aspects of the frame effect and enhance our understanding of how the visual system maintains spatial consistency in dynamic environments.

## Supplementary Material

Supplement 1

Supplement 2

Supplement 3
